# Collective effects of common SNPs in foraging decisions in *Caenorhabditis elegans* and an integrative method of identification of candidate genes

**DOI:** 10.1038/srep16904

**Published:** 2015-11-19

**Authors:** Zuobin Zhu, Qing Lu, Junjing Wang, Shi Huang

**Affiliations:** 1 State Key Laboratory of Medical Genetics, School of Life Sciences, Xiangya Medical School, Central South University. 110 Xiangya Road, Changsha, Hunan 410078, China

## Abstract

Optimal foraging decision is a quantitative flexible behavior, which describes the time at which animals choose to abandon a depleting food supply. The total minor allele content (MAC) in an individual has been shown to correlate with quantitative variations in complex traits. We have studied the role of MAC in the decision to leave a food lawn in recombinant inbred advanced intercross lines (RIAILs) of *Caenorhabditis elegans*. We found a strong link between MAC and the food lawn leaving rates (Spearman r = 0.4, *P* = 0.005). We identified 28 genes of unknown functions whose expression levels correlated with both MAC and leaving rates. When examined by RNAi experiments, 8 of 10 tested among the 28 affected leaving rates, whereas only 2 of 9 did among genes that were only associated with leaving rates but not MAC (8/10 vs 2/9, *P* < 0.05). The results establish a link between MAC and the foraging behavior and identify 8 genes that may play a role in linking MAC with the quantitative nature of the trait. The method of correlations with both MAC and traits may find broad applications in high efficiency identification of target genes for other complex traits in model organisms and humans.

*Caenorhabditis elegans* (*C. elegans*) is a good genetic model organism to study cellular and molecular mechanisms of nervous system development and function. Its nervous system represents its most complex organ with the most number of cells (302 neurons and 56 glial cells make up 37% of the somatic cells in a hermaphrodite) and the largest cellular diversity (at least 118 different neuron classes)^1^. Optimal foraging is an innate neurological trait central to evolutionary successes, and a key question in behavioral ecology is what determines an animal's choice between exploiting existing food and exploring other options that may lead to new resources^2^. Behavioral choice of suitable versus detrimental bacterial lawns is likely a key determinant of fitness in the worms^3^,^4^. *C. elegans* worms are more likely to leave lawns of low quality bacterial food^5^, but rarely leave a dense lawn of high-quality food^6^,^7^. This behavior is a quantitative trait linked with multiple genetic variations in natural populations^7^. Single nucleotide polymorphisms (SNPs) in two G protein-coupled receptors *npr-1*^8^,^9^ and *tyra-3*^7^ have been found to regulate the leaving rates. However, such major effect SNPs have yet to explain the quantitative characteristic of the leaving rate trait.

Complex traits are known to be polygenic and display quantitative genetic variations^10^. Current methods are focused on identifying individual loci with large effects on traits, including linkage analysis or quantitative trait loci (QTL) mapping and genome-wide association studies (GWAS)^11^,^12^. In most cases, using extremely large sample sizes is necessary for GWAS to detect weak effect loci^13^,^14^,^15^. Several ideas have been suggested to account for the missing heritability, including much larger numbers of variants of smaller effect^16^, pleiotropy^17^ –multiple phenotypes affected by the same genetic loci, ignoring genetic interactions^18^, and rarer variants^19^,^20^. In particular, epistatic interactions among alleles have been found to shape genomic diversity^21^,^22^. Given these more recently appreciated network-like complexities in genome structure and function, new approaches less influenced by the one gene one trait paradigm are needed to address the genetics of quantitative traits.

Single nucleotide polymorphisms (SNPs) typically have just two alleles and the minor allele (MA) has population frequency <0.5. Recent studies show a link between the total genome-wide minor allele contents (MAC) of an individual and numerous traits in model organisms, including life span, tumor susceptibility, learning and memory, sensitivity to alcohol and anti-psychotic drugs, and poor reproductive fitness^23^,^24^,^25^. MAC is defined as the number of MAs in an individual divided by the number of SNPs scanned. Higher MAC is more often linked with lower fitness and has also been linked with complex diseases such as Parkinson’s disease^25^,^26^. That one could find linkage between MAC and traits despite the likely presence of some MAs that are not related to any trait indicates the robustness of the MAC approach. Although certain MAs may be neutral and appear minor only because of co-segregation with deleterious alleles^27^ or background selection^28^, they may only represent noises and decrease the sensitivity of the MAC approach. While the MAC approach could be used for both natural populations and panels of inbreed lines of model organisms, the selective forces on the minor alleles are expected to be different between natural populations and inbred lines. The MAC approach is expected to work irrespective of the kinds of selection forces so long the MA is under negative selection.

The molecular mechanism of MAC action remains to be determined. Many genes have numerous expression QTLs (eQTLs)^21^,^29^,^30^,^31^. One straightforward hypothesis is that MAC may involve a collection of eQTLs that regulate a specific set of genes that in turn determine a trait. Such genes should have a certain type of correlation with both MAC and the trait affected by MAC. This double correlation could be used to more efficiently screen for candidate genes of complex traits relative to conventional approaches of only correlating with traits alone. RNAi and other approaches could then follow to confirm target genes^32^. In *C. elegans*, RNAi experiments are relatively easy to perform and simply involve feeding *E. coli* with dsRNA corresponding to the gene of interest^33^,^34^,^35^.

The genetic architecture of the leaving rate trait can be studied in recombinant inbred advanced intercross lines (RIAILs)^7^,^36^. The RIAILs are derived from *C. elegans* laboratory strain N2 (Bristol) and the natural isolate CB4856 (Hawaii or HW)^36^. The F1 up to F10 progenies are intercrossed to maximize random recombination and hence allelic diversity in the offspring population, which were then randomly selected for inbreeding up to 20 generations to generate the final panel of RIAILs homozygous for almost all variants or SNPs. During the random mating and subsequent inbreeding process, there are ample opportunities for harmful variants to be negatively selected, for beneficial variants to be positively selected, and for neutral variants to drift. Thus the frequencies of variants that exist in the established RIAILs panel are the results of both neutral drift and selection. The MAs are simply those that are present in less than half of the strains in a panel of RIAILs.

Certain known genetic factors could potentially affect SNPs frequency and hence a potential correlation between MAC and a trait in the RIAILs. There are laboratory-derived alleles with large effects^4^,^37^,^38^. Genetic incompatibility between HW and N2 strain could influence certain phenotypes^39^,^40^,^41^. If a trait is related to the incompatibility and hence linked to MAs selected by the incompatibility, its linkage with MAC may not necessarily be indirect since the trait may actually account for a part of the incompatibility. If certain minor alleles involved in a trait are positively selected by the incompatibility so to appear as major alleles, the linkage signal between the trait and MAC would be weakened, whereas if they are negatively selected by the incompatibility, they would remain as MAs and would not affect the MAC approach. If some MAs are only related to the incompatibility *per se*, they may not be related to any other traits and would be expected to weaken rather than enhance the association signals of any linkage between MAC and a trait not related to the incompatibility. Also, if some of these incompatibility-linked MAs are linked with a trait by chance and in fact account for the trait’s artificial linkage with MAC, one would expect an even stronger link between the trait and an index more determined by the incompatibility-related MAs, such as the HW allele content (HAC) defined as the number of HW alleles in a RIAIL strain divided by the number of SNPs scanned. Most MAs in the RIAIL panel are HW alleles, which may be related to the incompatibility^39^ and N2's laboratory adaptation favoring N2 alleles. Therefore, most of these broad scale non-specific selections on SNPs in RIAILs may increase the background noises to make it more challenging for the MAC approach to reveal a positive signal, and can be managed by simple tests to not to increase the likelihood of false positives.

We here employed the MAC approach to study the foraging behavior in RIAILs. We found a strong correlation between MAC and the leaving rates in the RIAILs. We established an integrative double correlation method for identification of target genes for the foraging behavior that requires candidates to be linked with both MAC and the leaving rates. We then carried out RNAi experiments to confirm a high success rate of the method.

## Results

### *npr-1* dependent MAC effects on the food lawn-leaving rates

**Figure 1:**
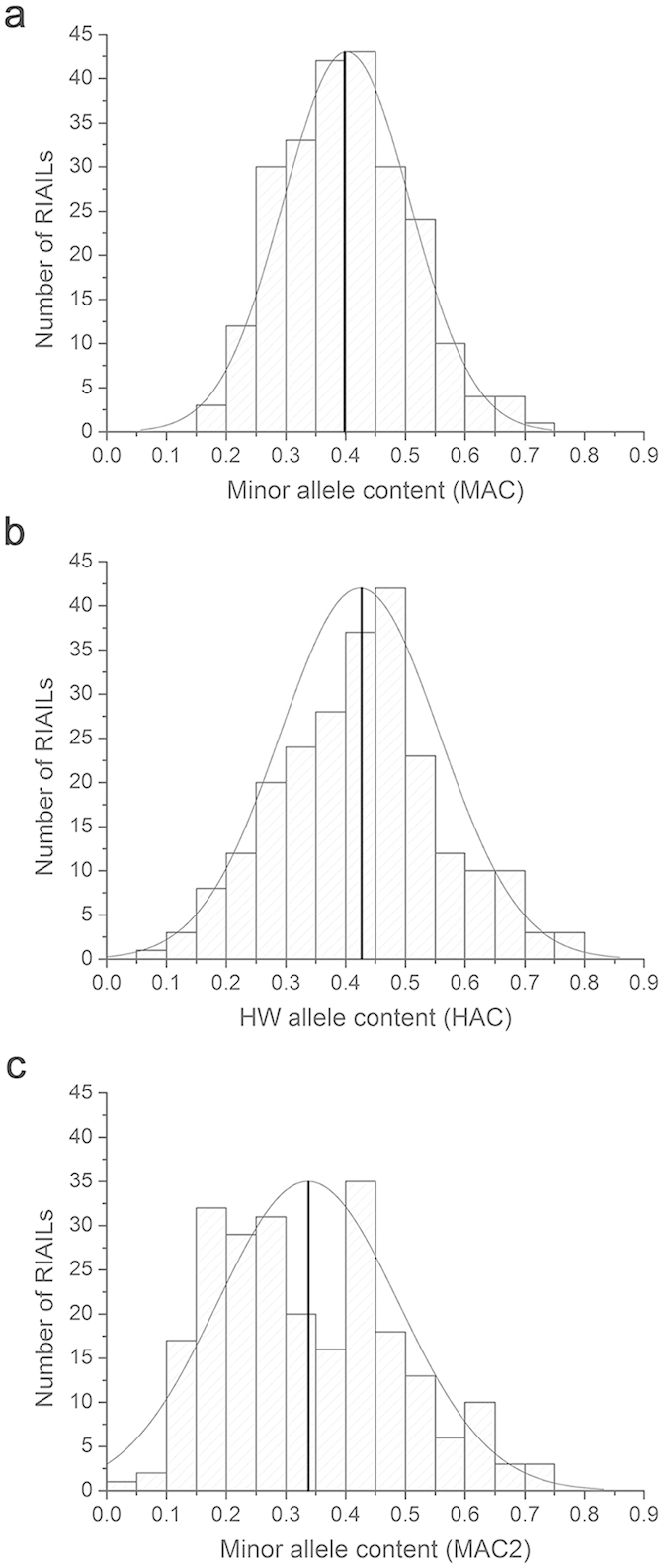
Distribution profiles of MAC and HAC in a panel of 237 RIAILs. (**a**) Minor allele content (MAC) calculated using 1454 SNPs. (**b**) HW allele content (HAC) calculated using 1454 SNPs. (**c**) MAC2 calculated using 526 SNPs with MAF significantly smaller than 0.5. The bold vertical line indicates the position of the mean value.

The 237 RIAILs used here have 1454 genotyped nuclear SNP markers span 98.6% of the physical length of the chromosomes^42^. We used these SNPs to calculate MAC of each RIAIL where MAs have MAF < 0.5 in the RIAILs panel or are present in less than or equal to 118 strains. We further calculated a more restricted MAC, designated MAC2, by using only those SNPs (a total of 526) that have MAs with MAF significantly smaller than 0.5 (*P* < 0.05, Chi-squared test). We also calculated the HW allele content (HAC) of each RIAIL which was defined as the number of HW alleles divided by the number of SNPs examined. We found a great variation in MAC (~0.17 to ~0.8), MAC2 (~0.03 to ~0.8), and HAC (~0.09 to ~0.8) among the RIAILs (Fig. 1 and Supplementary Table S1). The average MAC value of the 237 RIAILs was smaller than the average HAC value (0.40 vs 0.42, *P* = 0.04, One-way ANOVA), indicating that these two indexes may not be similar.

**Figure 2:**
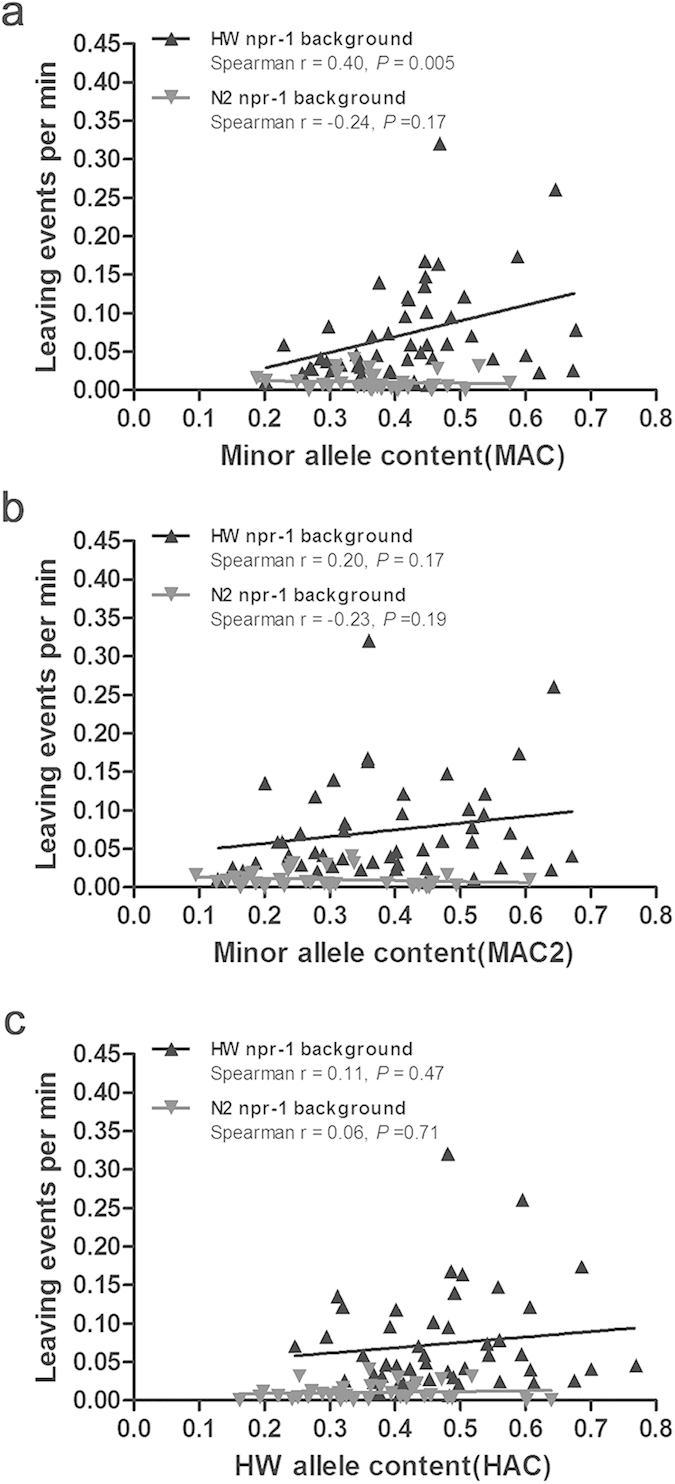
Correlations between leaving events and MAC or HAC. Correlations between MAC (**a**), MAC2 (**b**), or HAC (**c**) and the leaving rates in HW or N2 *npr-1* background.

To determine whether MAC may correlate with the food-lawn leaving rates, we examined 48 RIAILs with HW *npr-1* genotype and 35 with N2 *npr-1* genotype whose leaving rates have been previously published (Fig. 2a)^7^. Their parental strains HW and N2 differ in *npr-1* gene by one major effect SNP (F215V). We found that MAC was linked with higher food-lawn leaving rate in RIAILs with HW *npr-1* allele but not those with N2 *npr-1* allele (Fig. 2a). For the same set of RIAILs, there were no significant relationships between the leaving rates and MAC2 (Fig. 2b) or HAC (Fig. 2C). These results suggest a relationship between MAC and the leaving rates, which may involve many MAs with MAF close to 0.5. The lack of correlation with HAC indicates that any incompatibility between N2 and HW genomes or any laboratory conditions favoring N2 alleles may not account for the MAC-leaving rate linkage.

The above result could potentially be due to a few major effect SNPs that happen to associate with both MAC and leaving rates. Using 83 RIAILs for QTL analysis by PLINK, we found 834 SNPs linked with MAC, 958 with HAC, 601 linked with both MAC and HAC, and 4 SNPs linked with leaving rates, and 0 SNP linked with both leaving rates and MAC or both leaving rates and HAC (*P* < 0.05, FDR < 0.05) (Supplementary Table S2). These results suggest that the MAC-leaving rate association may not be accounted for by a few individual SNPs.

### Identification of novel genes for the food lawn-leaving rates

A gene affecting MAC as well as a complex trait should be linked with both MAC and the trait. Genes that satisfy such double correlation should have a higher probability to be real target genes than those with only single correlation. We next searched for genes whose expression profiles correlated with MAC alone, the leaving rates alone, and both MAC and the leaving rates. We analyzed gene expression profiles in the 48 RIAILs with HW alleles of *npr-1* using published datasets of normalized gene expressions^21^. We found 330 genes linked with MAC (Supplementary Table S3) and 197 genes with leaving events (Supplementary Table S4) and 28 genes with both MAC and leaving events among 15888 genes examined (*P* < 0.05, FDR < 0.15) (Table 1). Given these empirically observed rates of linkage with MAC (330/15888) and with leaving rates (197/15888), the number of genes being linked by chance to both MAC and leaving rates could be calculated as 15888 × (330/15888 × 197/15888) or 4.1. This number is seven times smaller than what was actually observed (28), indicating that genes linked to MAC had a higher than random chance of being linked to leaving rates and vice versa. This is not unexpected if the MAC-leaving rates relationship is real and specific.

There were 17 genes that were positively linked with both MAC and leaving rates and 11 that were inversely linked among the 28 associated with both MAC and leaving rates (Table 1). No gene was found to be positively linked with MAC but negatively linked with leaving rates. RNAi knockdown of the positively linked genes are expected to decrease the leaving rates while the reverse is expected for the inversely linked genes. Except *fbxa-103* that was picked due to its being ranked first among the positively linked genes, we arbitrarily picked from the 28 genes 6 positively linked genes (*ZC239.14, lnp-1, F42G2.2, R03H10.6, pho-1, and AC8.7*) and 3 inversely linked genes (*nspd-7, C25H3.1, F53C3.13)* for further confirmation and RNAi analyses.

We first determined that the correlations of these 10 genes with MAC may not be mostly due to some artifacts in the gene expression dataset. It has been reported that sequence polymorphisms cause many false cis eQTLs^43^. Probes used for hybridization analysis of gene expression could be affected by SNP-related mismatches. This could be a problem for the RIAILs as the N2 and HW strains were remarkably divergent in genome sequences^22^,^44^,^45^. Since MAC may include potential eQTLs, the link of MAC with mRNA expression of a gene could be affected by false eQTLs. To address this, we made use of the database WormQTL (http://www.wormqtl.org)^46^,^47^ and the expression profile data of RIAILs from the published literature^21^. We examined the number of eQTLs for each of the above 10 selected genes. Eight of these were found to have large number of eQTLs (6-152) while two had none (Supplementary Table S5). We examined by BLASTN the 10 tested genes for divergence between N2 and HW in the probes used for expression profile analysis by making use of the published N2^48^ and HW genome sequences^22^. We found no differences between N2 and HW for the 10 gene probes except for *ZC239.14* where there were 2 mismatches between N2 and HW (Supplementary Table S5). Therefore, most of the candidate genes (9/10) here are expected to have authentic mRNA expression profiles.

**Figure 3:**
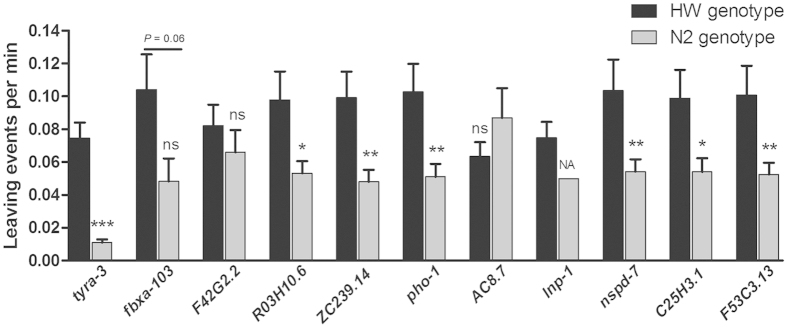
Polymorphisms in the tested genes and leaving events in RIAILs. Polymorphic alleles closely linked to *tyra-3* and the 10 candidate genes selected from the 28 genes doubly correlated with both MAC and the leaving rates were used to separate the 48 RIAILs into two groups of either carrying the HW or N2 allele of each gene. The average leaving rate of each group is shown as mean ± S.E.M. ns: non-significant, na: not applicable. *P < 0.05, **P < 0.01, ***P < 0.001, Student’s t-test, two-tailed.

Given the known allele specific effect of *tyra-3* or *npr-1* on the leaving rates, one may expect a target gene to show a high chance of allelic effect on the trait. We next determined whether any of these 10 genes may have polymorphic alleles that could differently affect the leaving rates. For each gene, we selected a SNP from the 1454 SNPs set that is closest to the gene in physical distance and hence most likely to co-segregate with the gene (see Table 1 for a list of these SNPs). Six of the 10 genes were found to exhibit allelic effects on the leaving rates in the 48 RIAILs with HW *npr-1* genotype, including *ZC239.14, R03H10.6, pho-1, nspd-7, C25H3.1*, and *F53C3.13* (Fig. 3). In contrast, only 214 of 1454 SNPs examined by QTL mapping showed association with leaving rate in the 48 RIAILs (*P* < 0.05, no FDR cutoff). All 6 SNPs/genes with allelic effects as shown in Fig. 3 were also found in those 214 SNPs identified by QTL mapping, indicating a significant enrichment in trait-associated genes/SNPs by using the double correlation method (214/1454 vs 6/10, *P* < 0.01). The result represents an independent piece of evidence for the roles of these 6 genes in the leaving rates.

**Figure 4:**
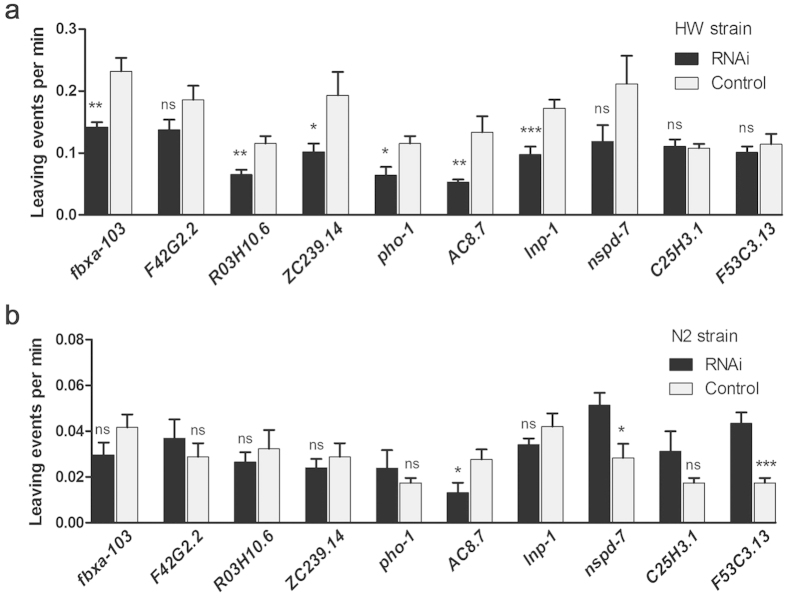
RNAi knockdown in the HW or N2 strain of 10 selected genes linked with both leaving events and MAC. (**a**) Data in the HW strain. (**b**) Data in the N2 strain. Data are means ± S.E.M. ns: non-significant, *P < 0.05, **P < 0.01, ***P < 0.001, Student’s t-test, two-tailed.

We next carried out RNAi experiments in the HW and N2 strain to further confirm the functions of the 10 selected genes. As might be expected, knockdown of most of the positively correlated genes decreased the leaving rates, mostly in the HW but not N2 strain (*ZC239.14, R03H10.6*, and *pho-1, lnp-1, fbxa-103,)*; only one positively correlated gene decreased the leaving rate in both HW and N2 strains (*AC8.7*) upon knockdown (Fig. 4). Knockdown of two of the three inversely correlated genes (*nspd-7* and *F53C3.13*) increased the leaving rate in N2 but not HW strain (Fig. 4).

**Figure 5:**
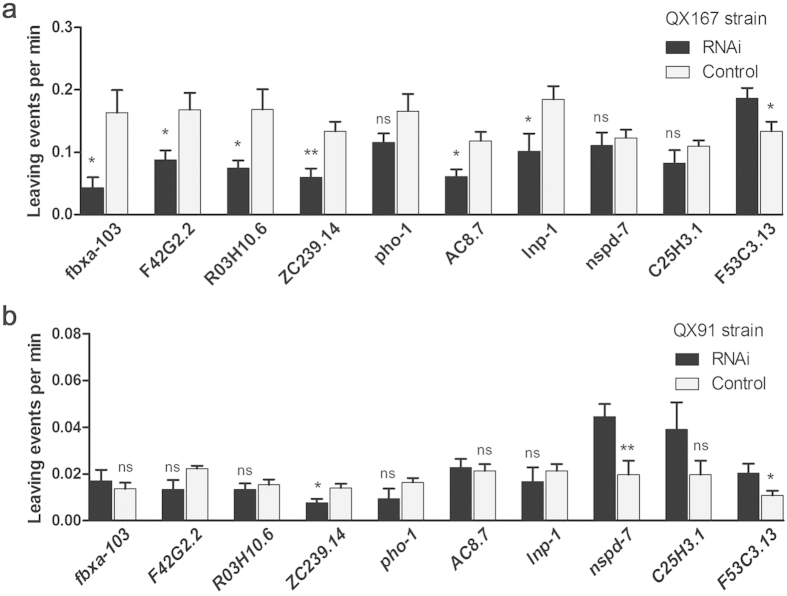
RNAi knockdown in RIAIL strains of 10 selected genes linked with both leaving events and MAC. (**a**) Data in the high MAC strain QX167. (**b**) Data in the low MAC strain QX91. ns: non-significant, *P < 0.05, Student’s t-test, two-tailed. Data are mean ± S.E.M.

We also performed RNAi experiments in two selected RIAIL strains. As more HW alleles were minor ones in the RIAILs, a high MAC strain may be expected to be more like the HW strain. Indeed, of the 6 positively correlated genes with a knockdown effect in the HW strain, all also showed expected knockdown effects mostly in the high MAC strain QX167 (Fig. 5). Also consistently, knockdown of the 3 inversely correlated genes in the low MAC strain QX91 showed similar results as above found in N2 strain (Fig. 5). All of the 6 positively correlated genes have high mRNA levels (at the top 25% among the 48 RIAILs) in QX167 strain but not in QX91 strain. Two of the inversely correlated genes (*nspd-7* and *F53C3.13*) had high mRNA levels (top 25%) in QX91 but not QX167 (Supplementary Table S6). Together, these experiments confirmed 8 target genes involved in leaving events, five of which also showed allelic effects.

To compare the double correlation approach with the single correlation method, we randomly selected 10 genes (6 positively linked: *K08C7.1*, *dos-2*, *Y25C1A.13*, *F44G4.7*, *F37H8.3*, *Y44A6C.1* and 4 inversely linked: *K01A11.3*, *amx-2*, *T16H12.2*, *ifa-1*) for RNAi among genes that were only associated with the leaving events but not MAC (Supplementary Fig. S2 and Supplementary Table S4). One of these genes (*ifa-1)* was non-suitable for analysis as its knockdown caused growth rate reduction and larval lethality in both HW and N2. Of the other 9 genes, two affected leaving rates upon knockdown in HW strain, including the positively correlated *K08C7.1* and the inversely correlated *K01A11.3.* The *K01A11.3* knockdown result of lower leaving rate was unexpected and could be due to indirect effects. None of the 9 genes affected leaving events upon knockdown in N2 strain. Thus, the success rate of this single correlation method (2/9) was significantly lower than that of the double correlation approach (8/10) (*P* =  0.01). We also found that one of these 9 genes (*Y25C1A.13*) showed allelic effects on leaving rates (Supplementary Table S7), which was lower than that found by the double correlation method (1/9 vs 6/10, *P* < 0.05, Fisher’s exact test, one tailed). As a further comparison, we randomly selected 5 genes for RNAi analyses in the HW and N2 strains from genes not correlated with either MAC or leaving events. None of these genes showed a knockdown effect on leaving events (Supplementary Fig. S2).

## Discussion

Our results revealed a connection between the collective effects of SNPs or MAC and the food lawn-leaving behavior of *C. elegans*. Although most MAs in the RIAILs were HW alleles, HAC showed no relationships with leaving rates. Thus, simply having more HW genome as indicated by HAC may not be sufficient to gain a higher leaving rate even though HW has higher leaving rates than N2. This also suggests that MAs related to laboratory conditions or the incompatibility between N2 and HW may not be related to the leaving rate trait.

The nearly linear correlation between MAC and leaving events suggests that the quantitative nature of the trait could be potentially accounted for by the additive effects of multiple weak effect MAs. Individual major effect SNPs such as *npr-1* and *tyra-3* as found previously may best account for an all or none phenomenon. The MAC concept may explain in general the quantitative characteristic of most complex traits. It addresses the role of the vast majority of common SNPs left untouched by the presently popular methods. That the MAC-leaving rate relationship could facilitate efficient identification of target genes, as independently verified by allelic effects and functional assays, indicates strongly that it is non-trivial. While there is no data or *a priori* reason for the relationship to be anything but causal, it would be challenging to obtain direct experimental evidence, as the hallmark of complexity or network may be the breakdown of causality^49^.

The potential reasons for a MA to be minor in a RIAIL panel include both drift and natural selection. Natural selection appears to be the major reason given the results here and elsewhere^23^. Both drift and natural selection could result in MAF not significantly different from 0.5. If a MA is both deleterious and beneficial with the deleterious effects slightly stronger than the beneficial effects, then the MA should be expected to have MAF close to 0.5. Examples of genetic variations being both deleterious and beneficial may include those involved in immunity and reproduction, where high level variations may be beneficial to adaptive immunity but deleterious to reproduction^23^. Therefore, one would expect MAC2 to be less informative than MAC because MAC2 fails to take into account of MAs with MAF close to 0.5 that may be under both negative and positive selections. Indeed, our results showed no correlation between MAC2 and the leaving rate trait.

Variations originate from recombination and random mutations. An individual should have an optimal amount (a Pareto optimum) of variations or randomness/entropy in the molecular building blocks with either too high or too low amounts both reducing fitness^23^. At optimal equilibrium, a minor allele is more likely to be slightly more deleterious or under more negative selection than a major allele. To a highly ordered biological system, all random variations have a deleterious aspect in terms of random noises or entropy and may hence be under negative selection, if not individually then collectively. Thus, of the two alleles of a SNP, the one under more negative selection, i.e., the minor allele, is likely to be associated with higher levels of noises/entropy. A trait is the end outcome of multiple highly ordered molecular pathways and should be subject to influence by the noises in the molecular building blocks.

Most SNPs may not have large effects individually but a group of them together over a threshold limit may have significant effects. The observation of higher MAC linkage with mostly lower rather than higher fitness suggests that the reason for most MAs to be at slightly lower frequency is that they may be under balancing selection with slightly more negative selection^23^,^25^,^26^. The results here further confirm this notion and showed a more meaningful MAC when MAs were simply defined as those with MAF < 0.5 relative to when MAs were called only for those with MAF significantly smaller than 0.5. While one might expect MAs with MAF close to 0.5, especially in a relatively small panel of RIAILs, to be likely non-related to selection, they may also contain many that are under nearly even balancing selection. Although the relative abundance of MAs under balancing selection may need to be more precisely determined, that we only observed a meaningful MAC by including those MAs with MAF close to 0.5 indicates balancing selection for most MAs.

Most cellular components exert their functions through interactions with other components, and this network of interactions is not random but is characterized by a set of organizing principles^50^. Consistent with such principles, traits linked with MAC usually have more QTLs^24^, and individual QTLs often affect multiple genes^51^. Many genes are known to each have numerous eQTLs^21^,^29^,^30^. In the case of aging, for example, the gene network integrity declines with age with ~75% of all genes affected (P < 0.0001)^31^. Given the genome wide scale, MAC action is likely through gene networks involving the collective effects of multiple loci. Consistently, we did not find any individual loci that could be linked with both MAC and leaving rates. In fact, we consistently found MAC and major effect mutations to be two largely independent ways of negatively impacting a trait. The MAC-leaving rate relationship was only found in RIAILs carrying the *npr-1* HW allele but not the N2 allele. So, MAC could act without involving genetic changes in *npr-1* and vice versa. Similar relationships between MAC and major effect mutations such as *kras2* have been previously found in lung cancers in mice^25^.

Why worms with more minor alleles have a tendency to leave the food lawn? We can speculate as to how randomness in the DNA building parts might promote higher leaving rates. A faster leaving rate means a more constant in and out of the food lawn for no apparent reasons, indicating a more random kind of behavior. If the leaving behavior is in part a stochastic process, a worm with more noises within its system would be expected to show a more random phenotype such as more frequent fluctuations between opposite ends of a behavior. Also, leaving a food resource to explore unknown options is a high risk or costly behavior, which may explain the slightly more negative selection on the alleles responsible for higher leaving rates.

Knockdown of genes that were positively correlated with both MAC and leaving rates mostly decreased the leaving rates, while the reverse was found for the inversely correlated genes, as expected. But the former result was mostly found in the HW and a high MAC strain but not the N2 strain while the later found in the N2 and a low MAC strain but not HW strain. This is likely due to the difference in the leaving behavior between these two types of strains with HW or the high MAC strain having higher leaving rates. Knockdown of a gene that was positively linked with leaving rate would be expected to decrease the leaving rate, which could be more obviously observed in a strain that has high leaving rate. On the other hand, knockdown of a gene that was inversely linked with leaving rate would be expected to increase the leaving rate, which could be more easily observed in a strain that has low leaving rate. In addition, our results could be affected by differences in RNAi sensitivity^33^,^34^,^35^. RNAi in N2 is highly effective, but in HW RNAi sensitivity for germline expressed genes has been lost^33^. However, since we did observe positive RNAi results for most of our tested genes in the HW strain, the low sensitivity did not appear to be significant, perhaps because the expressions of these genes in non-germline tissues were more important for the leaving rate trait.

Of the 28 candidate genes identified by the double correlation method and of the 8 confirmed by RNAi, most have unknown functions (Table 1). Of note, *fbxa-103* encodes an F-box motif and an FTH/DUF38 motif, both predicted to mediate protein-protein interactions and are unusually common in the worm genome (~200 genes). It will be interesting to determine if genes related to MAC may be enriched in functions related to network and complexity.

Candidate genes can be identified by several methods, including prior knowledge of the biological pathway, linkage studies, comparative genomics strategy, and GWAS^52^. However, the false positive rate is generally high for most genome wide approaches. Combining different strategies can increase the power to identify candidates^53^,^54^. Few of these approaches, however, are meant to identify genes responsible for the quantitative characteristic of a complex trait. We have established a new integrative method for screening decision-making genes or genes for complex traits in general. The method of double correlation with both MAC and leaving rates was significantly more effective than merely correlating gene expressions with the trait alone. Genes identified by this method were highly enriched in true targets as shown by both allelic effects and RNAi. Previously known genes involved in leaving rates such as *npr-1* and *tyra-3* were not found correlated with MAC. Therefore, the method is a good complement to existing methods. It appears more biased toward genes with more eQTLs as 8 of 10 examined genes have multiple eQTLs (merely ~900 genes in *C. elegans* were found to have an eQTL^31^), which is not unexpected as the MAC index should involve numerous eQTLs. As the MAC index is linked with numerous traits and diseases in model organisms and humans^23^,^24^,^25^,^26^, the double correlation method may greatly facilitate future identification of targets genes for these traits and diseases.

## Materials and Methods

### MAF, MAC, and HAC calculation

The calculation of MAF and MAC was done as previously described^26^. HW allele content (HAC) in a RIAIL was calculated as the number of HW alleles carried by the RIAIL divided by the number of SNPs examined. The SNP datasets were obtained from public databases and literature^42^. The MAF of each SNP in the panel of RIAILs was calculated by PLINK^55^. From such MAF data, we obtained the MA set, which excluded non-informative SNPs with MAF =  0 or 0.5 in the panel. The MA set was equivalent to the genotype of an imagined individual who is homozygous for all the MAs. The MAC of each RIAIL was then determined by matching the genotype of a RIAIL with the MA set; the number of identical genotypes was scored as the number of MAs for the RIAILs^23^. The MAC of a strain was calculated by dividing the number of MAs carried by the strain by the number of total SNPs scanned.

### Statistical methods

Spearman correlations were performed using GraphPad Prism5. The correlation between gene expression and leaving events or MAC were analyzed using the Significance Analysis of Microarrays (SAM) software with 1,000 sample permutations. SAM uses permutations to estimate the false discovery rate (FDR) and an adjustable threshold allows for control of the FDR^56^. SAM adopts q-value as the lowest FDR at which the gene is called significant. Here we used the most stringent criteria as defined by SAM to call a gene significant, which are treating data as quantitative type, 1000 permutations, and KNN value 10 (K-nearest neighbor). In order to minimize missing true positives, we set the FDR cut-off value at 15%, *P* < 0.05 (Spearman). The differences in leaving events between RNAi and control treated worms were examined by Student’s t test, two tailed.

### SNPs association analysis

The PLINK software package (v1.07) with the quantitative trait association option was used to search for SNPs linked with MAC and HAC. The quantitative trait association method uses a permutation procedure (permuting genotype rather than phenotype) to control for the non-independence of individuals. The analysis of phenotype-genotype association is a standard regression of phenotype on genotype that ignores family structure^55^. Q-value estimation for false discovery rate control was done using R package 'qvalue'^57^.

### Strains and media

*C. elegans* RIAILs were gifts from L. Kruglyak. *C. elegans* were cultivated at 20°C on normal growth medium (NGM) and seeded with the *E. coli* OP50. We used 237 RIAILs for calculating MAC, MAC2, and HAC. Among these, 48 RIAILs with HW *npr-1* genotype were used to perform correlation studies of the leaving rate trait with both MAC/MAC2/HAC and gene expression patterns. In addition, 35 RIAILs with N2 *npr-1* genotype were used for correlation studies between the leaving rate trait and MAC, MAC2, and HAC. We used the combined 83 RIAILs for QTL mapping analysis with FDR < 0.05 (Supplementary Table S1). We also used the 48 RIAIL set for QTL mapping without FDR cutoff.

### Published data

Food lawn-leaving rate datasets were obtained from A. Bendesky, P. McGrath, and C. Bargmann. Normalized gene expression datasets for the RIAILs were from published datasets^21^.

### Screening candidate genes for foraging decisions

Normalized gene expression data are from published datasets^7^,^21^. RIAILs were cultured in the *E. coli* OP50 for gene expression analysis. RIAILs used for leaving rate experiments were cultured in *E. coli* HB101. We confirmed that the relative lawn-leaving rate of each RIAIL were similar in these two bacteria foods. Thus the correlation between gene expression and the leaving rate should not be affected by the different food conditions. We first selected the genes linked with leaving events by SAM at the threshold of FDR < 15%. We then determined the correlation between these genes and MAC. The genes linked with both leaving events and MAC were defined as candidates for foraging decisions.

### Analysis of behavior in the Leaving assays

Leaving assays was performed essentially as described^7^. Ten worms were transferred onto the 6 cm NGM plates which were seeded with 70 μL fresh *E. coli* OP50 (diluted in M9 buffer, OD600 = 0.1) (conditioning plate). The worms were acclimated on the conditioning plate for 30 min, followed by transferring 7 of them onto another plate seeded with 10 μL fresh *E. coli* OP50 (assay plate). After 1hr when the leaving events were stable, the number of leaving events was recorded manually by examining the video recordings. A leaving event was defined when the whole body of a worm left the bacterial lawn and the worm did not reverse immediately to return to the lawn. The leaving rate was calculated as the number of leaving events per worm minute spent inside the bacteria lawn. Experiments were repeated at least three times.

### RNAi assays

RNAi was performed essentially as described^58^. L1 worms placed on the NGM plates seeded with the HT115(DE3) bacteria were transformed with the L4440 vector containing a fragment corresponding to the target gene. The primers of RNAi fragments were listed in Supplementary Table S8. The RNAi fragments were amplified from genomic DNAs of the strain used in the RNAi experiment. The worms were cultured for 72 hours at 20 °C for RNAi to take effect. The adults were used for the leaving rate experiment. For negative controls, the L1 worms were fed with the HT115 bacteria carrying the L4440 vector with no gene fragment. For positive controls, the L1 worms were fed with the HT115 bacteria carrying the L4440 vector with *uaf*-1 fragment.

## Additional Information

**How to cite this article**: Zhu, Z. *et al.* Collective effects of common SNPs in foraging decisions in *Caenorhabditis elegans* and an integrative method of identification of candidate genes. *Sci. Rep.* **5**, 16904; doi: 10.1038/srep16904 (2015).

## Supplementary Material

Supplementary Information

Supplementary Dataset 1

Supplementary Dataset 2

Supplementary Dataset 3

## Figures and Tables

**Table 1:** 28 genes linked with both MAC and leaving rates and their nearest SNPs.

Gene	Direction^1^	SNP	CHR^2^	Distance^3^	Function^4^
dhs-3	+	UCE1-967	I	8928	oxidoreductase activity
fbxa-103	+	CE1-258	I	-11159	Cyclin-like F-box
F16G10.15	+	UCE2-1078	II	51086	unknown function
F42G2.2	+	UCE2-1078	II	-68042	unknown function
ZC239.5	−	UCE2-1259	II	41747	unknown function
ZC239.14	+	UCE2-1259	II	−32889	unknown function
btb-17	−	CE2-123	II	−22653	BTB/POZ-like, IBTB/POZ fold
btb-16	−	CE2-123	II	−24126	BTB/POZ-like, IBTB/POZ fold
Y46D2A.1	+	CE2-123	II	51686	IPR005071:Protein of unknown function DUF274,
F14D2.15	−	UCE2-1312	II	−53464	BTB/POZ-like, IBTB/POZ fold
F18A12.4	−	UCE2-1313	II	−6234	metalloendopeptidase activity; metallopeptidase activity
F19B10.10	−	UCE2-1456	II	−23560	unknown function
F53C3.13	−	UCE2-1515	II	−40130	catalytic activity
R03H10.6	+	CE2-130	II	14931	nucleic acid binding; double-stranded DNA binding
pho-1	+	UCE2-1610	II	exon	acid phosphatase activity nucleotide binding;
C25H3.1	−	CE2-141	II	−101566	protein kinase activity; protein serine/threonine kinase activity; ATP binding; transferase activity, transferring phosphorus-containing groups
btb-2	−	UCE2-1665	II	3313	BTB/POZ-like, IBTB/POZ fold
C39B5.10	+	CE3-117	III	−15412	unknown function
nspd-7	−	CE4-26	IV	−10312	unknown function
M7.8	+	CE4-174	IV	−10990	unknown function
W02B12.1	+	CE4-177	IV	−10118	hydrolase activity, acting on ester bonds
unc-31	−	UCE4-1194	IV	6220	calcium ion binding; phosphatidylinositol-4,5-bisphosphate binding; lipid binding; metal ion binding
F36F12.1	+	CE5-121	V	1004	integral to membrane; intrinsic to membrane,
W01A11.7	+	CE5-156	V	60562	unknown function
nhr-231	+	CE5-254	V	29776	DNA binding; sequence-specific DNA binding transcription factor activity; steroid hormone receptor activity; zinc ion binding; sequence-specific DNA binding; metal ion binding
AC8.7	+	UCE6-531	X	−118721	unknown function
lnp-1	+	UCE6-870	X	−17683	metal ion binding; locomotory behavior
spp-4	+	CE6-225	X	−3973	integral to membrane; intrinsic to membrane,

The genes are listed in the order of chromosome and chromosome position.

^1^Direction: Direction of correlation with both MAC and leaving rates. +, positive correlation; −, inverse correlation.

^2^Chr: Chromosome where the gene and SNP are located.

^3^Distance without a ‘–’ sign means that the SNP is located in front of the 5′ end of the gene and the distance is between the SNP and first codon of the gene. Distance with a ‘–’ sign means that the SNP is located at the 3′ end of the gene and the distance is between the SNP and the stop codon of the gene.

^4^Gene of protein function were annotated from GENE ONTOLOGY (GO) Consortium or InterPro resource.
