# Supplementary material for: Collective effects of common SNPs in foraging decisions in Caenorhabditis elegans and an integrative method of identification of candidate genes
Source: Sci Rep. 2015 Nov 19;5:16904. doi: 10.1038/srep16904 (PMC4652280; doi:10.1038/srep16904)
Supplement: Supplementary Information [file srep16904-s1.pdf]

SUPPLEMENTARY INFORMATION

Collective effects of common SNPs in foraging decisions in *Caenorhabditis elegans* and an integrative method of identification of candidate genes

Zuobin Zhu, Qing Lu, Junjing Wang, and Shi Huang*

State Key Laboratory of Medical Genetics, School of Life Sciences, Xiangya Medical School, Central South University. 110 Xiangya Road, Changsha, Hunan 410078, China

*Corresponding author: huangshi@sklmg.edu.cn

Supplementary Fig 1: RNAi knockdown in HW or N2 strain of genes linked with leaving events but not MAC. Data are mean ± S.E.M. ns: non-significant, * *P* <0.05, Student's t-test.

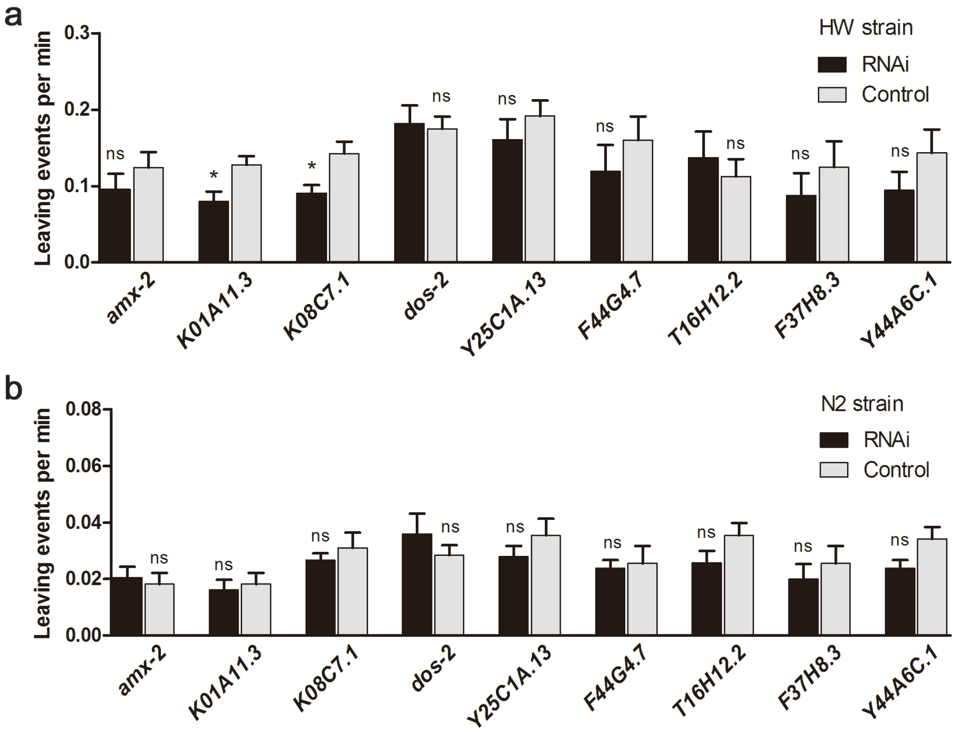

Supplementary Fig 2: RNAi knockdown in the N2 and HW strain of genes linked with neither leaving events nor MAC. Data are mean ± S.E.M. ns: non-significant.

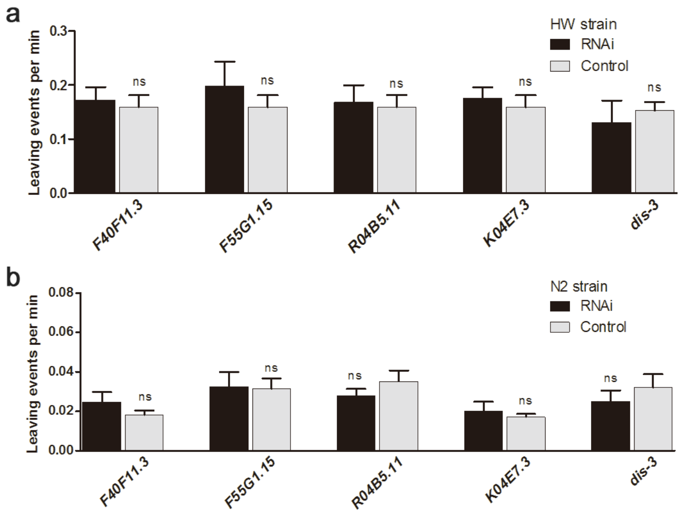

Supplementary Table S1: Basic description of the 83 RIAILs used in the study.

Strain	npr-1 genotype	tyra-3 genotype	HAC	MAC	MAC2	Leaving R	SEM
QX10	HW	HW	0.4451	0.4493	0.2274	0.0589	0.0295
QX107	HW	HW	0.4818	0.4859	0.5360	0.0949	0.0164
QX11	HW	HW	0.5072	0.4577	0.2900	0.0420	0.0164
QX12	HW	HW	0.4296	0.2701	0.2566	0.0289	0.0099
QX124	HW	HW	0.6076	0.4192	0.3937	0.0398	0.0053
QX125	HW	HW	0.4859	0.4460	0.3585	0.1677	0.0355
QX127	HW	HW	0.6747	0.6733	0.5624	0.0258	0.0154
QX13	HW	HW	0.5608	0.3918	0.4482	0.0247	0.0119
QX131	HW	HW	0.4811	0.2955	0.3199	0.0373	0.0109
QX132	HW	HW	0.4529	0.2729	0.3040	0.0275	0.0043
QX133	HW	HW	0.7698	0.6007	0.6028	0.0454	0.0216
QX14	HW	HW	0.4591	0.4481	0.5132	0.1022	0.0372
QX144	HW	HW	0.4477	0.4395	0.4429	0.0494	0.0199
QX149	HW	HW	0.3230	0.3024	0.1511	0.0256	0.0066
QX151	HW	HW	0.3508	0.2297	0.2201	0.0589	0.0133
QX154	HW	HW	0.2955	0.2983	0.3234	0.0832	0.0206
QX158	HW	HW	0.3863	0.3409	0.4025	0.0466	0.0061
QX167	HW	HW	0.5952	0.6460	0.6432	0.2609	0.0834
QX168	HW	HW	0.4364	0.5175	0.5764	0.0705	0.0068
QX169	HW	HW	0.6069	0.5065	0.5378	0.1216	0.0434
QX179	HW	HW	0.6864	0.5880	0.5905	0.1737	0.0760
QX182	HW	HW	0.4832	0.4502	0.4464	0.0063	0.0063
QX195	HW	HW	0.2467	0.3649	0.2548	0.0704	0.0221
QX196	HW	HW	0.5038	0.4667	0.3585	0.1639	0.0380
QX198	HW	HW	0.4979	0.3487	0.2812	0.0205	0.0048
QX199	HW	HW	0.4062	0.3938	0.3480	0.0228	0.0166
QX200	HW	HW	0.5581	0.4467	0.4798	0.1475	0.0314
QX202	HW	HW	0.4131	0.2577	0.1670	0.0220	0.0090
QX205	HW	HW	0.4023	0.3721	0.2777	0.0454	0.0077
QX208	HW	HW	0.6131	0.6213	0.6397	0.0228	0.0084
QX213	HW	HW	0.3924	0.4158	0.4112	0.0959	0.0173
QX223	HW	HW	0.3801	0.3430	0.1863	0.0318	0.0100
QX233	HW	HW	0.5938	0.4797	0.4728	0.0603	0.0085
QX24	HW	HW	0.5409	0.3897	0.3216	0.0738	0.0215
QX31	HW	HW	0.3122	0.4457	0.2007	0.1357	0.0379
QX34	HW	HW	0.4021	0.4213	0.2777	0.1181	0.0317
QX37	HW	HW	0.4811	0.4687	0.3603	0.3209	0.1110
QX39	HW	HW	0.4914	0.3759	0.3058	0.1400	0.0608
QX42	HW	HW	0.5436	0.4241	0.5185	0.0593	0.0004
QX45	HW	HW	0.4227	0.2866	0.2373	0.0415	0.0190

QX58	HW	HW	0.7010	0.5498	0.6714	0.0409	0.0096
QX61	HW	HW	0.3678	0.3168	0.3662	0.0331	0.0167
QX64	HW	HW	0.3678	0.3939	0.4014	0.0238	0.0084
QX65	HW	HW	0.5601	0.6770	0.5185	0.0786	0.0184
QX86	HW	HW	0.4085	0.4292	0.5211	0.0114	0.0043
QX91	HW	HW	0.3753	0.2021	0.1283	0.0112	0.0078
QX98	HW	HW	0.3196	0.4199	0.4130	0.1217	0.0514
QX108	HW	N2	0.4893	0.3478	0.4042	0.0303	0.0055
QX121	N2	N2	0.1979	0.2502	0.1722	0.0112	0.0032
QX128	N2	N2	0.3003	0.2893	0.1265	0.0071	0.0031
QX129	N2	N2	0.3168	0.3649	0.2004	0.0000	0.0000
QX134	N2	N2	0.1622	0.2680	0.1634	0.0000	0.0000
QX157	N2	N2	0.2722	0.2021	0.1547	0.0117	0.0062
QX165	N2	N2	0.3251	0.4598	0.4587	0.0057	0.0057
QX171	N2	N2	0.4811	0.4536	0.4288	0.0037	0.0037
QX174	N2	N2	0.3780	0.3643	0.1793	0.0175	0.0015
QX176	N2	N2	0.3629	0.3684	0.1828	0.0186	0.0077
QX178	N2	N2	0.1931	0.3526	0.2917	0.0052	0.0030
QX181	N2	N2	0.4082	0.4206	0.2970	0.0047	0.0047
QX185	N2	N2	0.2426	0.3622	0.2302	0.0016	0.0016
QX187	N2	N2	0.3141	0.4117	0.2232	0.0090	0.0033
QX192	N2	N2	0.3595	0.3787	0.3005	0.0019	0.0019
QX193	N2	N2	0.3361	0.4048	0.4271	0.0000	0.0000
QX209	N2	N2	0.6398	0.5076	0.4523	0.0000	0.0000
QX210	N2	N2	0.4550	0.3615	0.2636	0.0070	0.0016
QX212	N2	N2	0.1931	0.3430	0.3093	0.0038	0.0038
QX221	N2	N2	0.2213	0.3794	0.2707	0.0060	0.0060
QX32	N2	N2	0.5179	0.5289	0.3374	0.0314	0.0210
QX38	N2	N2	0.4263	0.3643	0.4797	0.0160	0.0070
QX43	N2	N2	0.4175	0.4202	0.4366	0.0028	0.0028
QX47	N2	N2	0.6021	0.4564	0.4482	0.0000	0.0000
QX49	N2	N2	0.3608	0.3375	0.3339	0.0405	0.0124
QX52	N2	N2	0.4450	0.4807	0.3873	0.0056	0.0056
QX55	N2	N2	0.2515	0.3725	0.2285	0.0038	0.0038
QX56	N2	N2	0.4048	0.3608	0.2953	0.0293	0.0118
QX62	N2	N2	0.4880	0.3945	0.4938	0.0023	0.0023
QX68	N2	N2	0.3203	0.1883	0.0949	0.0158	0.0069
QX70	N2	N2	0.3945	0.5759	0.6063	0.0096	0.0048
QX71	N2	N2	0.2674	0.2990	0.1863	0.0048	0.0048
QX73	N2	N2	0.2889	0.3177	0.1424	0.0083	0.0018
QX74	N2	N2	0.4711	0.4649	0.2408	0.0278	0.0158
QX77	N2	N2	0.2536	0.3100	0.2443	0.0319	0.0250
QX82	N2	N2	0.4331	0.3119	0.2362	0.0215	0.0052

Supplementary Table S2: Potential major effect SNPs linked with MAC, HAC, or leaving rates. (attached as separated Excel files)

Supplementary Table S3: Genes linked with MAC. (attached as separated Excel files)

Supplementary Table S4: Genes linked with leaving events. (attached as separated Excel files)

Supplementary Table S5: The number of eQTLs for each of the 10 tested candidate genes doubly correlated with both MAC and leaving rates. The table also shows identities between N2 and HW in these genes and probes used for these genes.

Gene	Probe	Position	eQTLs	Identities (gene)	Identities (probe)
fbxa-103	A_12_P119163	chrI:14295999-14295940	31	0.99	1
ZC239.14	A_12_P112216	chrII:3221471-3221412	115	0.75	0.97
AC8.7	A_12_P109825	chrX:231170-231279	152	1	1
lnp-1	A_12_P118204	chrX:4590079-4590138	0	1	1
F53C3.13	A_12_P110917	chrII:3921681-3921622	132	0.99	1
C25H3.1	A_12_P101781	chrII:5696760-5696701	51	1	1
nspd-7	A_12_P113469	chrIV:10268975-10269187	0	0.99	1
R03H10.6	A_12_P116652	chrII:4171878-4171819	144	0.99	1
pho-1	A_12_P112324	chrII:5233429-5233293	6	0.99	1
F42G2.2	A_12_P119326	chrII:2425192-2425251	70	0.99	1

Supplementary Table S6: mRNA levels of the 10 tested genes in RIAILs.

			48 HW RIAILs							
			Mean	SE	95% CI of mean	Median	25% Percentile	75% Percentile	Min	Max
**fbxa-103**	NA	1.39	0.18	0.15	-0.81, 1.53	0	-0.25	0.72	-0.99	1.97
**F42G2.2**	-1.06	0.98	0.06	0.08	-0.71, 0.95	-0.01	-0.29	0.45	-1.13	1.51
**R03H10.6**	-2.26	2.37	0.27	0.43	-3.26, 2.74	1.75	-2.89	2.33	-3.59	3.07
**ZC239.14**	NA	0.83	0.21	0.18	-1.24, 1.60	0.71	-1	1.17	-1.97	1.95
**pho-1**	-0.61	0.75	0.04	0.05	-0.33, 0.42	0.02	-0.2	0.34	-0.64	1.03
**AC8.7**	-0.9	1.15	-0.01	0.12	-0.90, 1.06	-0.12	-0.7	0.55	-0.99	1.53
**lnp-1**	-0.41	1.09	0.08	0.04	-0.22, 0.36	0.05	-0.1	0.23	-0.41	1.09
**nspd-7**	2.08	-4.03	0.16	0.34	-2.75, 2.80	1.22	-2.39	2.32	-4.03	3.41
**C25H3.1**	0.44	-1.43	0.03	0.10	-0.92, 0.87	0.19	-0.51	0.53	-1.43	1.06
**F53C3.13**	1.38	-0.64	-0.11	0.18	-1.70, 1.28	0.21	-1.29	1.06	-2.59	1.8

Supplementary Table S7. Allele specific effects of the 9 genes identified by the single correlation method. SNPs nearest to these genes are listed. Each SNP was used to separate the 48 RIAILs into two groups of either carrying the HW or N2 allele of each gene. The difference in the average leaving rates between the two groups was examined by Student's t test (two tailed) and the *P* values of the test are shown.

Gene Name	Direction[1]	SNP	CHR[2]	Distance[3]	*P* value (T.TEST)[4]
amx-2	-	CE1-209	I	20861	0.901094
K01A11.3	-	UCE2-1313	II	49455	0.483471
K08C7.1	+	UCE4-1116	IV	-3924	0.910193
dos-2	+	UCE2-1515	II	-8252	0.073865
Y25C1A.13	+	UCE2-1230	II	16093	0.042772
F44G4.7	+	CE2-170	II	-2927	0.131773
T16H12.2	-	CE3-185	III	7841	0.179695
F37H8.3	+	UCE2-1985	II	77811	0.963926
Y44A6C.1	+	UCE5-3397	V	-62506	0.177455

[1]Direction: Direction of correlation with both MAC and leaving rates. +, positive correlation; -, inverse correlation.

[2]Chr：Chromosome where the gene and SNP are located.

[3] Distance without a '–' sign means that the SNP is located in front of the 5' end of the gene and the distance is between the SNP and first codon of the gene. Distance with a '–' sign means that the SNP is located at the 3' end of the gene and the distance is between the SNP and the stop codon of the gene.

[4] The difference in the average leaving rate between N2 and HW allele groups was examined by Student's t test (two tailed) and the P values of the test are shown.

Supplementary Table S8: Primers for RNAi fragments.

Chr	Gene Name	Fwd Primer Seq	Rev Primer Seq
IV	*K08C7.1*	CGCTTTGCCTGTGTCTCATA	TGCTGCTTTCAATCGTTTTG
II	*dos-2*	ACTGTACTCGGAGGGGACCT	GATCCGGGTTTTGTCTGAAA
IV	*M7.8*	AGATTTCATCGCAATCACACTTT	TTCAGTCGTCCAAAATCTTTCAT
III	*K01A11.3*	TCGTGTGGAGCAAAACTGAG	GAAAAATGGGGACATTGTGG
IV	*dis-3*	TGATGACGAGAACGATGAGC	AACGTCAATGCTCCATTTCC
I	*fbxa-103*	AAGGCGGGATCAAAAACTCT	CACCAAACAACTTGACACCG
X	*lnp-1*	GAAAACGTATGTGGCGTGTG	ACTCCGGGACCTTCTCATTT
II	*ZC239.14*	GGAATACTTCTCAATGAACGCAC	AACAACAATTTGGTAAGGCTGAA
II	*F42G2.2*	CTCAACTCCGTAGGCACACA	GTAACGTCGGGTTCCAAAGA
IV	*nspd-7*	AGACGAATTGCAACATAGGAAAA	ACAGAAATCGCCAGTTTATCAGA
V	*R04B5.11*	GAATTTCAGAGTTGAGTCGGATG	AATAGGCAAGTATGGAGTGAGCA
IV	*F40F11.3*	TCCCTTTTTACACTCTGATCCAA	CCATACATCGATGAGGACAAAAT
IV	*F55G1.15*	CAGCTCTCTCCTCTGCACCT	CCAGCATTATCATTCCCCAC
X	*K04E7.3*	CGTTAACTTCCGACCACGAT	CTCCATGGGTAACCAGCCTA
II	*R03H10.6*	GCCTCGAGCATTGTATCCAT	AGCGACGACTATCCCAGCTA
II	*pho-1*	ACTTCCGTCTTGCTCGTGAT	GTCCAACTTGCTCGGACAAT
II	*C25H3.1*	CCCTTTTTGGATAGAATTATGGC	ACACAATATCACAAGTGAACCCC
IV	*AC8.7*	TCCGTATTTTTACAGCGAATTGT	GAAAAAGAAACGGAAGAAAGGAA
II	*Y25C1A.13*	TTGCCTCACTCCAGAATACATTT	ATTCTCGGTCATTTTTCGAATTT
II	*F44G4.7*	CCTTTTCCCACTCGTTTTCA	TTGGTTTTAGGTCCTGCGTC
III	*T16H12.2*	ATTTCCCTCTACCTCGTTGATTC	GAGAAGAAGGAAACACTTTGGGT
V	*Y44A6C.1*	AAATCAAATCCCGGAAAACC	TGACTTCAGCACCATCAAGC
II	*F37H8.3*	GCAGACACACTTAGCTGCCTATT	CTTTTCTGCAGCTTCAATGTTTT
I	amx-2	TAAAAATGGTTGAGGCGCTATTA	ATTGCTTCATTTTCAGGATCTCA
II	*F53C3.13*	GGTTTTTCTTCAAATTTCGCTTT	TCGTGGTTTCTACTGTGATGATG
III	*uaf-1*	TGCAACTCGGCGACAAGCAG	AGAATTGACGATTGTGGTACTTG
